# Supplementary material for: Transfer of sulfur and chalcophile metals via sulfide-volatile compound drops in the Christiana-Santorini-Kolumbo volcanic field
Source: Nat Commun. 2024 Jun 11;15:4968. doi: 10.1038/s41467-024-48656-9 (PMC11167051; doi:10.1038/s41467-024-48656-9)
Supplement: Supplementary file 1 — Supplementary information [file 41467_2024_48656_MOESM1_ESM.pdf]

Transfer of sulfur and chalcophile metals via sulfide-volatile compound drops in the Christiana-Santorini-Kolumbo volcanic field

Clifford Georges Charles Patten[1*], Simon Hector[2], Stephanos Pantelis Kilias[3], Marc Ulrich[4], Alexandre Peillod[2], Aratz Beranoaguirre[2,5], Paraskevi Nomikou[3], Elisabeth Eiche[2,6], Jochen Kolb[2]

[1]*Institute of Mineralogy and Petrography, University of Innsbruck, Austria*

[2] *Chair for Geochemistry and Economic Geology, Institute of Applied Geosciences (AGW), Karlsruhe Institute of Technology, Germany*

[3]*Department of Geology and Geoenvironment, National and Kapodistrian University of Athens, Greece*

[4]*Institut Terre et Environnement de Strasbourg, Université de Strasbourg, CNRS, France*

[5]*Institut für Geowissenschaften, Goethe-Universität Frankfurt, Germany*

[6]*Laboratory of Environment and Raw materials Analysis (LERA), AGW, Karlsruhe Institute of Technology, Germany*

**email: clifford.patten@uibk.ac.at*

Supplementary Discussion, Supplementary Figures 2,3,4,5 and 6

Supplementary Discussion: Comparison between sulfide bleb and sulfide ovoid and calculation of emanation coefficients

Supplementary Figure 1: Comparison of trace metal composition of magmatic sulfide blebs and recalculated magmatic sulfide liquid

Supplementary Figure 2: Magmatic sulfide blebs and associated vesicles in plagioclase phenocrysts within andesitic enclaves

Supplementary Figure 3: Overview of variably oxidized sulfide ovoids from Kameni and Kolumbo volcanic rocks

Supplementary Figure 4: Different type of magnetites present in the CSK

Supplementary Figure 5: Comparison of metal content in magmatic sulfides and mineralized samples from Kolumbo diffusers

Supplementary Figure 6: Micro-XRF maps of Al, Ca, Cu, Fe, K, Mg, S, Si and Ti (TH-20-30-A)

Supplementary Figure 7: Mineral proportion maps calculated from micro-XRF elemental maps for sample TH-20-30-A

Supplementary Figure 8: Micro-XRF maps of Al, Ca, Cu, Fe, K, Mg, S, Si and Ti (TH-20-30-2)

Supplementary Figure 9: Mineral proportion maps calculated from micro-XRF elemental maps for TH-20-30-2

Supplementary Discussion

Comparison between sulfide bleb and sulfide ovoid compositions

The magmatic sulfide bleb composition is assumed to be representative of the primary magmatic sulfide liquid while the pyrrhotite and chalcopyrite analyzed in the ovoids are representative of differentiated sulfide phases such as MSS and ISS[1,2]. Direct comparison between the sulfide bleb and the sulfide ovoid composition is not possible and a primary magmatic sulfide composition needs to be calculated:

$$C_{x\,sulf} = C_{x\,Po} * X_{Po} + C_{x\,Cpy} * X_{Cpy} + C_{x\,SMag} * X_{SMag} \quad \text{(S1)}$$

with $C_{x\,Po}$, $C_{x\,Cpy}$ and $C_{x\,SMag}$ the concentration of the element *x* in the pyrrhotite, chalcopyrite and magnetite which have crystallized from the initial magmatic sulfide liquid and X_{Po}, X_{Cpy} and X_{SMag} the mass fraction of pyrrhotite, chalcopyrite and magnetite which have crystallized from the magmatic sulfide liquid. The pyrrhotite and chalcopyrite concentrations are from the analysis of ovoids (this study) while the magnetite concentrations are from Dare et al.[3] (sample MRC11). Magnetite crystallization from a sulfide liquid is common[1–4] and needs to be taken into account for the calculations. Trace element data of magnetites which crystallized from a sulfide liquid are scarce and the data from Dare et al.[3], although from a radically different magmatic environment, are the closest proxy found for the CSK system. The primary magmatic sulfide liquid is calculated assuming different proportions of pyrrhotite (0.85-0.55), chalcopyrite (0.1-0.4) and magnetite (0.05; Fig. 1).

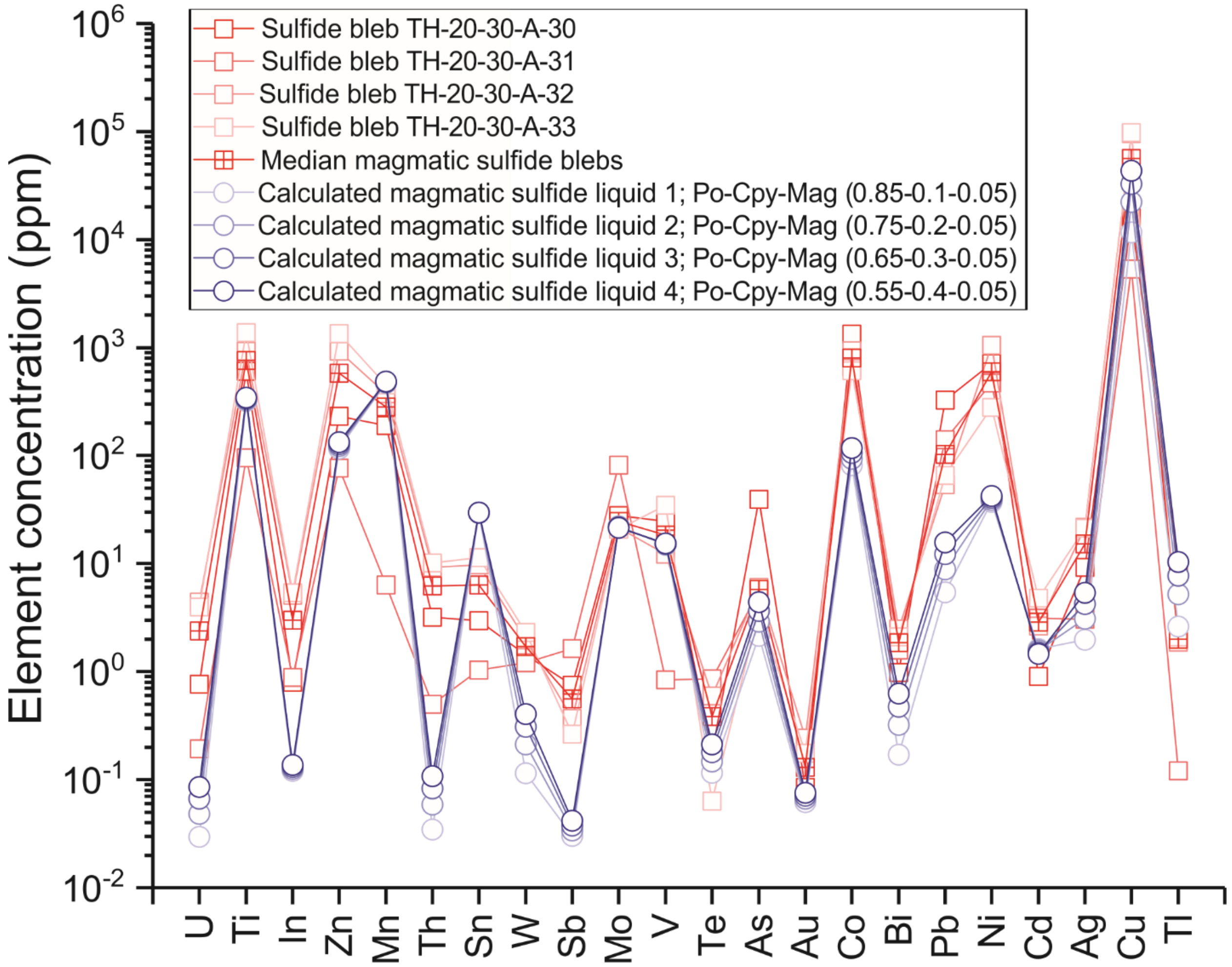

Supplementary figure 1.Comparison of trace metal composition of magmatic sulfide blebs and recalculated magmatic sulfide liquid with different proportions of pyrrhotite, chalcopyrite and magnetite.

The calculated primary magmatic compositions and the magmatic blebs have very similar element concentrations and profiles despite some differences for a few elements (U, In, Th and Sb). The overall good correlation between the two groups highlights the genetic link between the magmatic sulfide blebs and the sulfide ovoids.

Calculation of emanation coefficients

The emanation coefficients [5,6], although developed for melts and post-eruptive lavas, can be applied to the compound drop system and are defined as:

$$\varepsilon_x = (C_{x\,sulf} - \frac{M_{mag}}{3 * M_{sulf}} * C_{x\,mag})/C_{x\,sulf} * 100 \qquad \text{(S2)}$$

The calculation of the emanation coefficients requires characterizing the concentration of any given element *x* within the whole primary magmatic sulfides as defined in the equation (S1). The molar mass of the primary magmatic sulfide can be defined as:

$$M_{sulf} = \frac{1}{X_{Po}/\mathrm{M_{Po}} + X_{Cpy}/\,\mathrm{M_{Cpy}} + X_{SMag}/\mathrm{M_{SMag}}} \qquad \text{(S3)}$$

with $\mathrm{M_{Po}}$, $\mathrm{M_{Cpy}}$ and $\mathrm{M_{SMag}}$ the molar mass of pyrrhotite, chalcopyrite and magnetite which have crystallized from the sulfide liquid.

The emanation coefficients are then expressed in percent as:

$$\varepsilon_x = \frac{((C_{x\,Po} * X_{Po} + C_{x\,Cpy} * X_{Cpy} + C_{x\,SMag} * X_{SMag}) - \frac{\mathrm{M_{Mag}}}{3*(\frac{1}{X_{Po}/\mathrm{M_{Po}} + X_{Cpy}/\,\mathrm{M_{Cpy}} + X_{SMag}/\mathrm{M_{SMag}}})} * C_{x\,Mag})}{(C_{x\,Po} * X_{Po} + C_{x\,Cpy} * X_{Cpy} + C_{x\,SMag} * X_{SMag})} * 100 \qquad \text{(S4)}$$

It is important to emphasise that the $C_{x\,Mag}$ and $\mathrm{M_{Mag}}$ are the element concentration and the molar mass of the magnetite replacing the magmatic sulfides after oxidation while $C_{x\,SMag}$, $\mathrm{M_{SMag}}$ and X_{SMag} are the element concentration, the molar mass and the fraction of the magnetite which crystallizes from the sulfide liquid during sulfide liquid differentiation. Some element analyses in magnetite and pyrrhotite show concentrations below the limit of detection (see Supplementary Data 5). These data are not discarded as they yield important information on element distribution within the sulfide-volatile compound drops. Half the values of the limit of detections are used for the calculations as they are likely more representative of the true concentrations than the limit of detections. The emanation coefficients are calculated for variable fractions of pyrrhotite, chalcopyrite and magnetite in the primary magmatic sulfides and are presented in the Supplementary Data 3 and Fig. 2c. Importantly, the emanation coefficients can have negative values, because the compound-drop system is not a closed system and can interact with the silicate melt. Elements compatible in magnetite can partition into the magnetite from the silicate melt during sulfide oxidation; on the other hand, strongly chalcophile and volatile elements are not considered to partition into the magnetite.

Supplementary Figures

Supplementary Figure 2

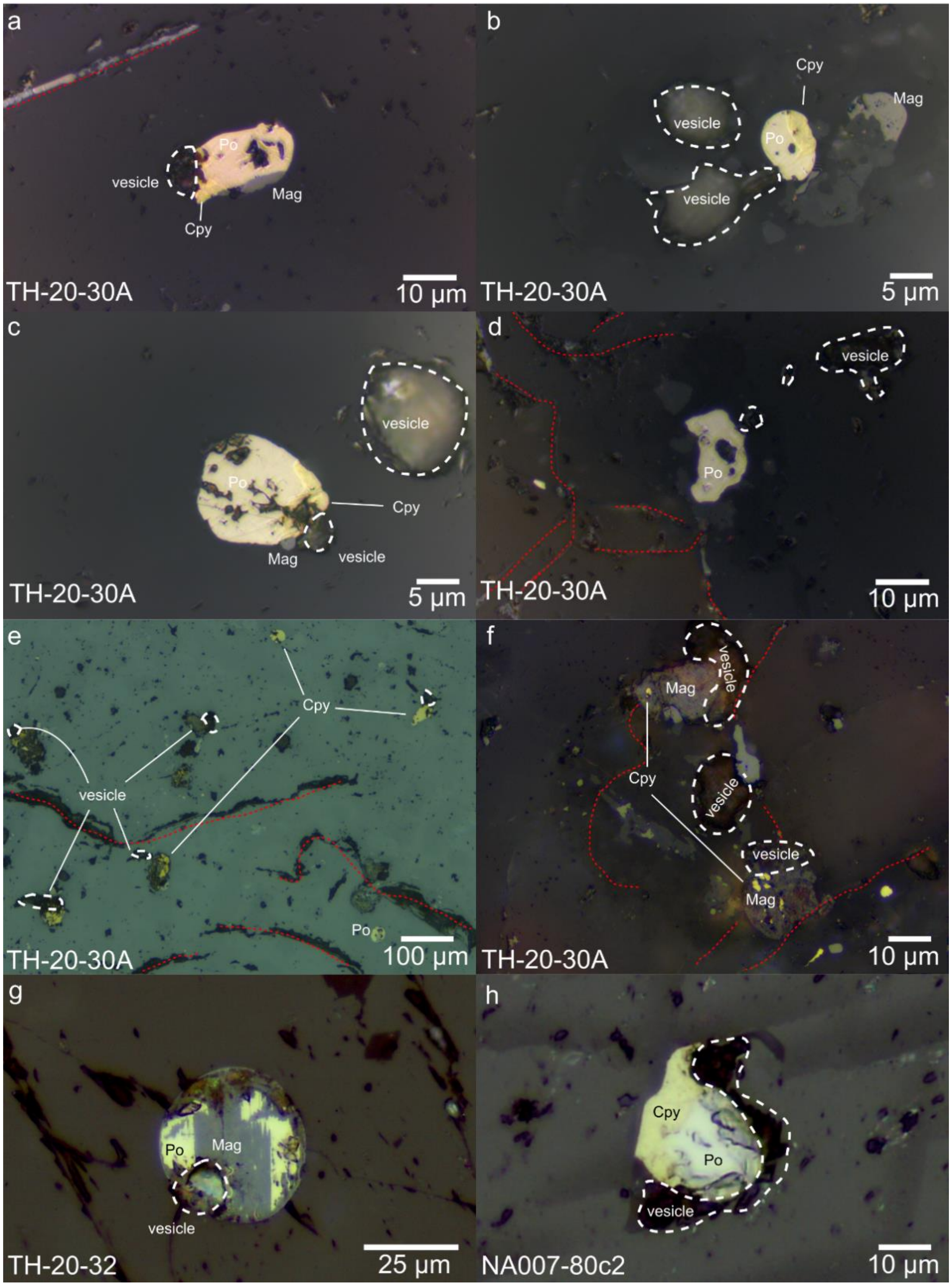

Supplementary figure 2. Magmatic sulfide blebs and associated vesicles in plagioclase phenocrysts within andesitic enclaves. a-b-c-d-h) Fresh magmatic sulfide blebs composed of pyrrhotite (Po), chalcopyrite (Cpy) and minor magnetite (Mag) associated with vesicles. e) Fresh and altered sulfide blebs. f-g) Extensively and partially oxidized magmatic sulfide blebs replaced by magnetite. Vesicles can still be observed. Red dot lines are fractures.

Supplementary Figure 3

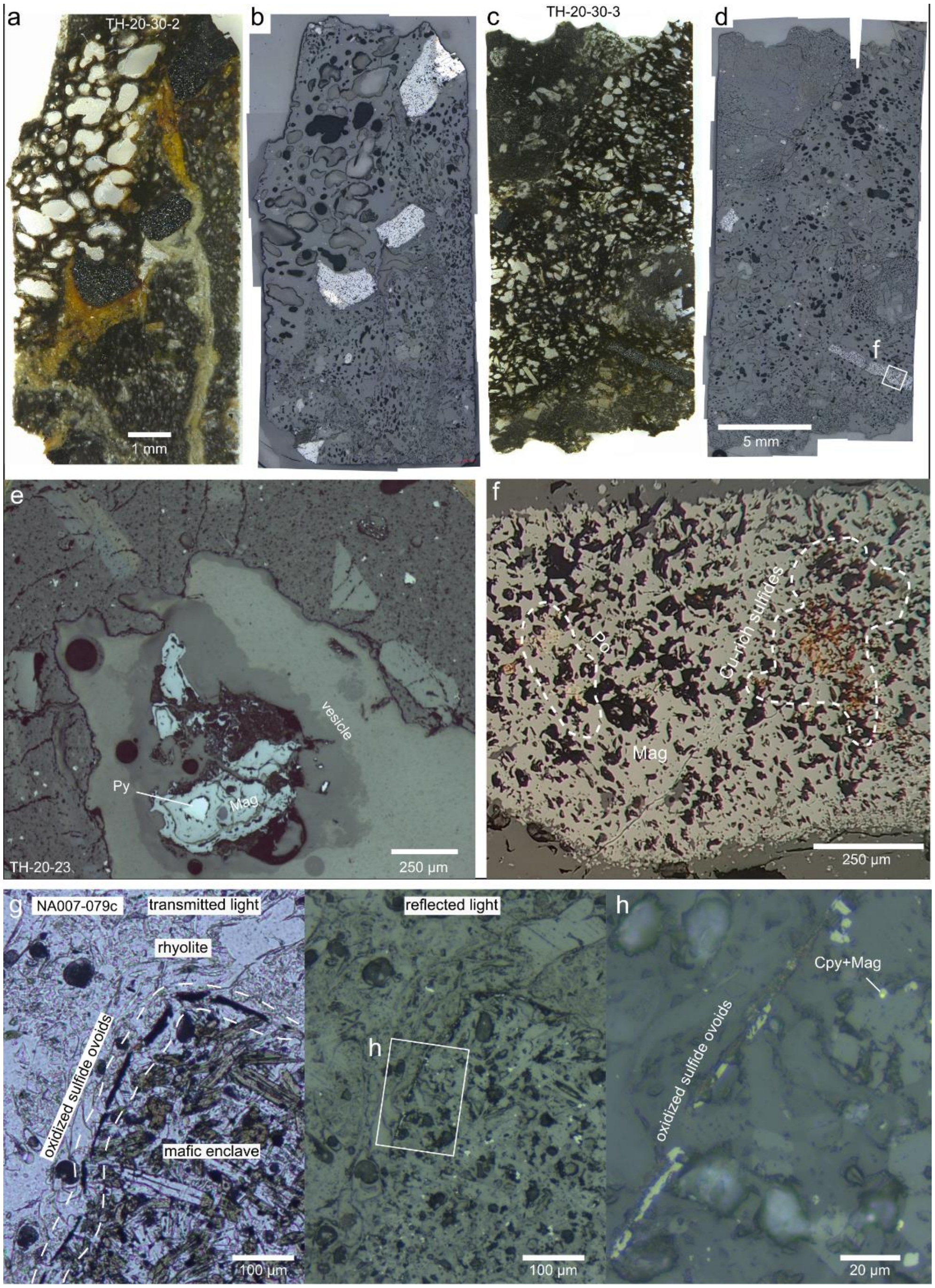

Supplementary figure 3: Overview of variably oxidized sulfide ovoids from Kameni and Kolumbo volcanic rocks. a) and c) polished thin section scans showing the andesitic and dacitic melts transition zone. b) and d) reflected light mosaics showing the position of the variably oxidized ovoids. e) Fully oxidized sulfide ovoids within large vesicles. Discrete pyrite is preserved in the core. f) Detail from d) showing preserved interstitial pyrrhotite (Po) and chalcopyrite (Cpy) within porous magnetite (Mag). g) Fine lining of oxidized sulfide ovoid at a mafic-rhyolite interface from the Kameni volcano observed in transmitted and reflected light. h) close up of the oxidized sulfide ovoids at the enclave margin. In the enclave micrometric magmatic sulfides (Po and Cpy) and magnetites are ubiquitous.

Supplementary Figure 4

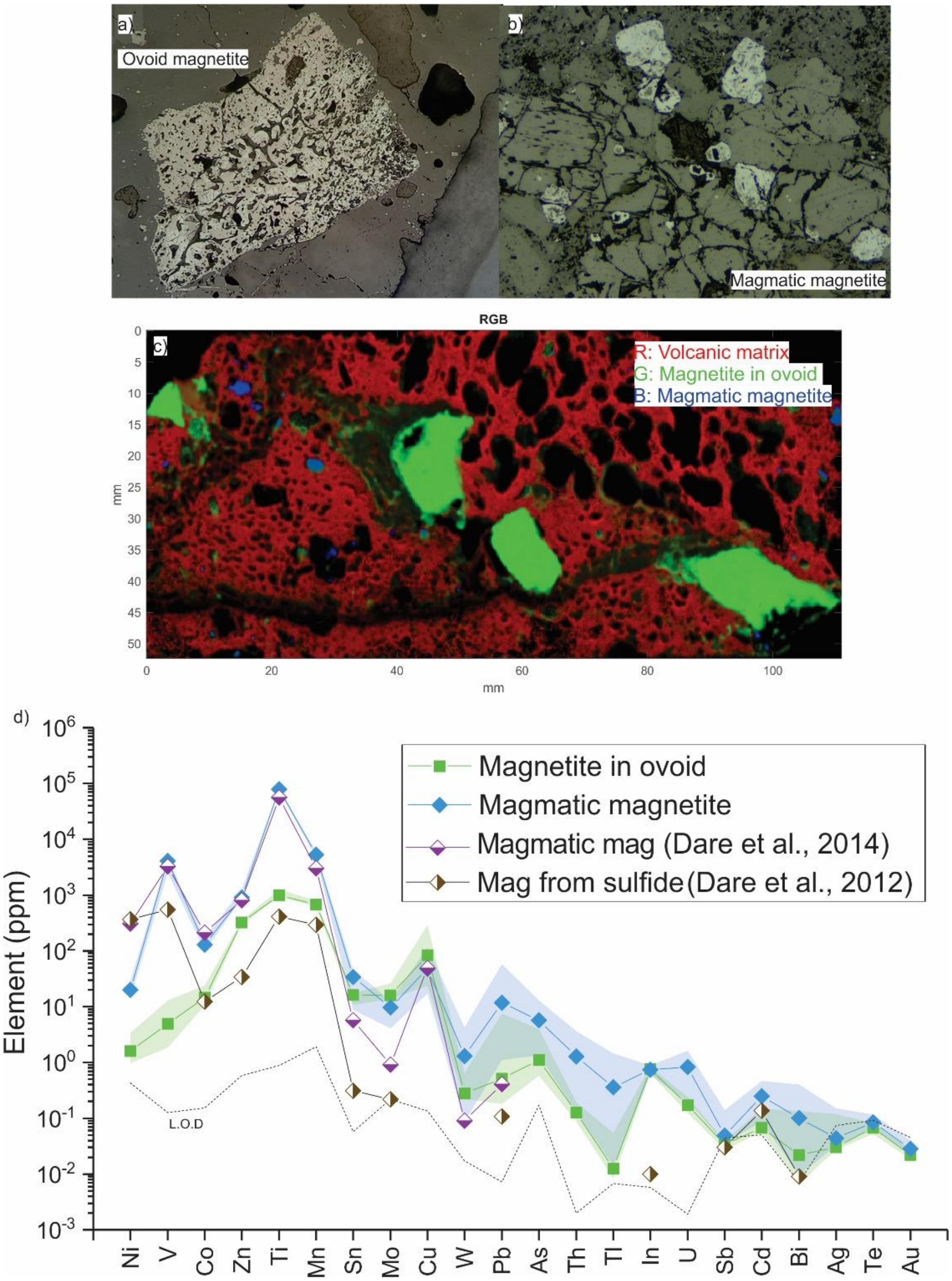

Supplementary figure 4. Different type of magnetites present in the CSK. a) Magnetite related to sulfide ovoid, b) magmatic magnetite found in mafic enclaves, c) micro-XRF map showing spatial relationship between the two magnetite types, magnetites related to the sulfide ovoids (a) are in green and the magmatic magnetite (b) are in blue, and d) Median trace element concentrations of magnetite in ovoids and magmatic magnetites. Shaded areas represent upper and lower quartile. Magmatic magnetite have similar metal concentrations than magmatic magnetite crystallized from a silicate melt[8]. Ovoid magnetite shows distinctive metal concentrations from both the magmatic magnetites which crystallized either from a silicate melt or a sulfide melt.

Supplementary Figure 5

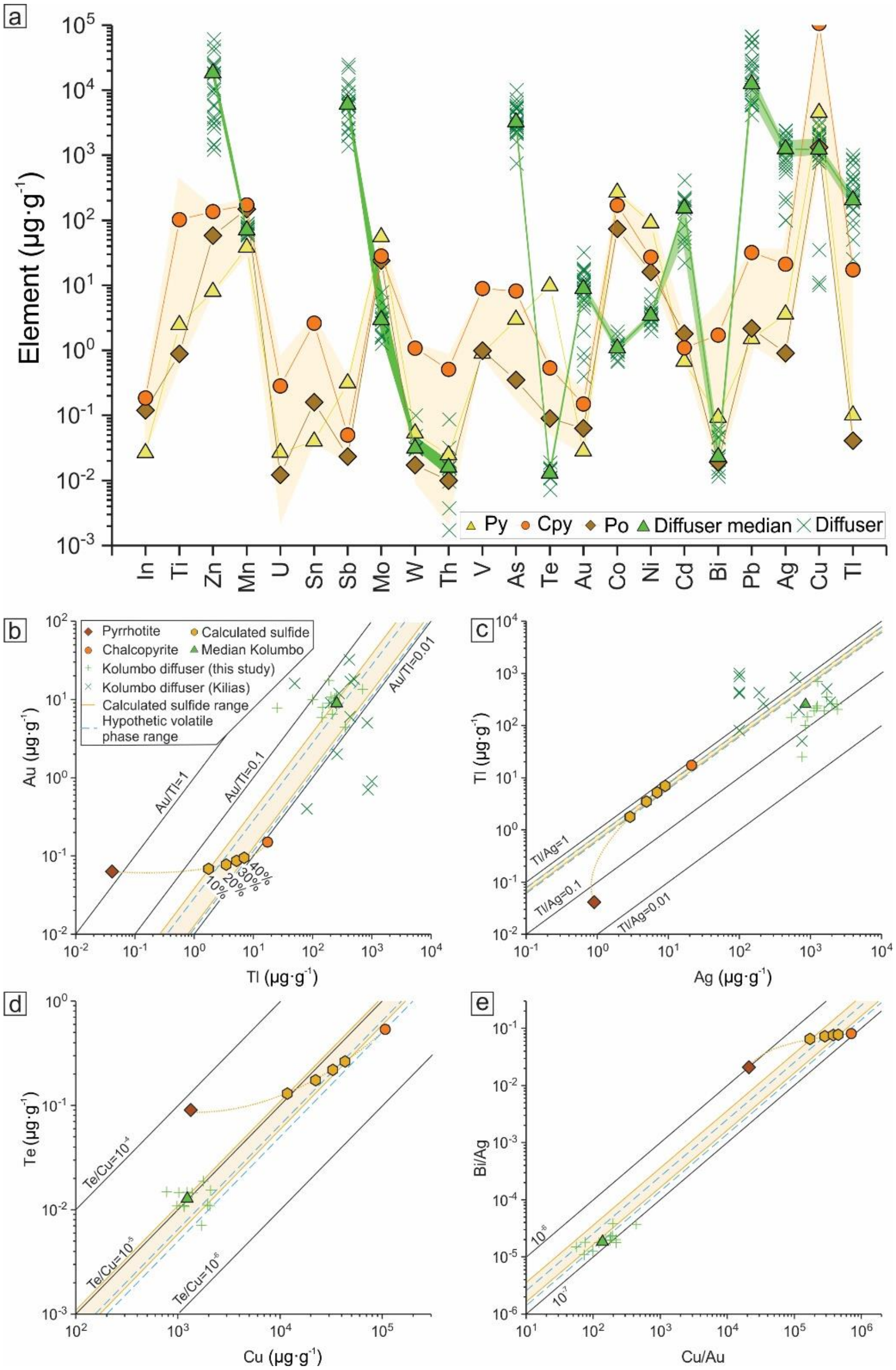

Supplementary figure 5: Comparison of metal content in magmatic sulfides and mineralized samples from Kolumbo diffusers, a) median concentrations for pyrite (Py), chalcopyrite (Cpy), pyrrhotite (Po) and Kolumbo diffuser, shaded areas represent the range of the upper and lower quartile, Kolumbo diffuser data are from Kilias et al.[59] and from this study (see Supplementary Data 6), b) Au vs Tl, c) Tl vs Ag, d) Te vs Cu and e) Bi/Ag vs Cu/Ag. The calculated magmatic sulfides are determined with various fractions of pyrrhotite and chalcopyrite (see Supplementary Discussion for details), the percentage represent the chalcopyrite proportion. The hypothetic volatile phase metal ratio range corresponds to the magmatic sulfide ratio range factorized by the emanation coefficients of the selected elements, see Fig. 4 and manuscript for details.

Supplementary Figure 6

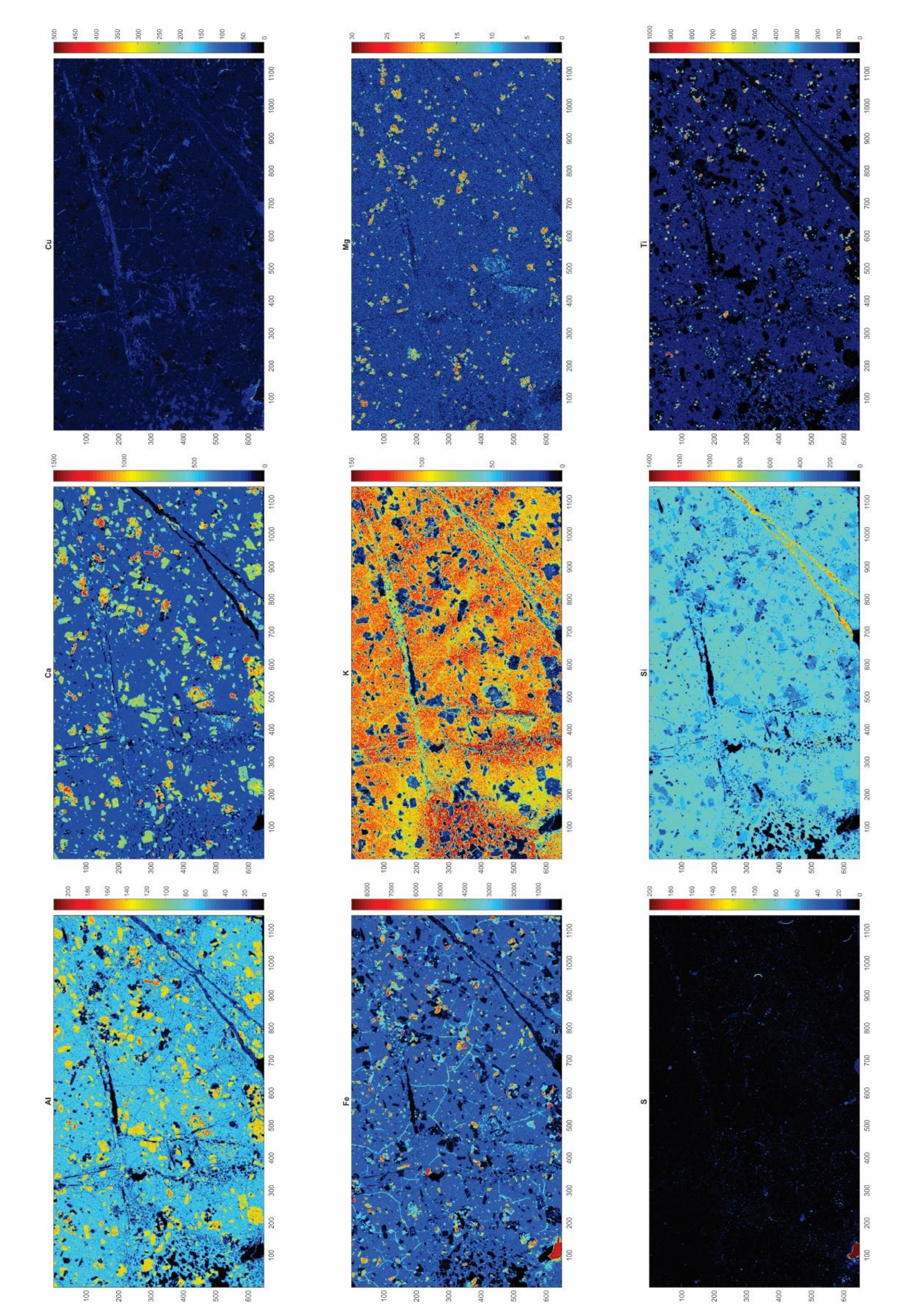

Supplementary figure 6. Micro-XRF chemical maps of Al, Ca, Cu, Fe, K, Mg, S, Si and Ti from sample TH-20-30-A used for calculation of phase proportions by selecting regions of interests. The scales are absolute counts.

Supplementary Figure 7

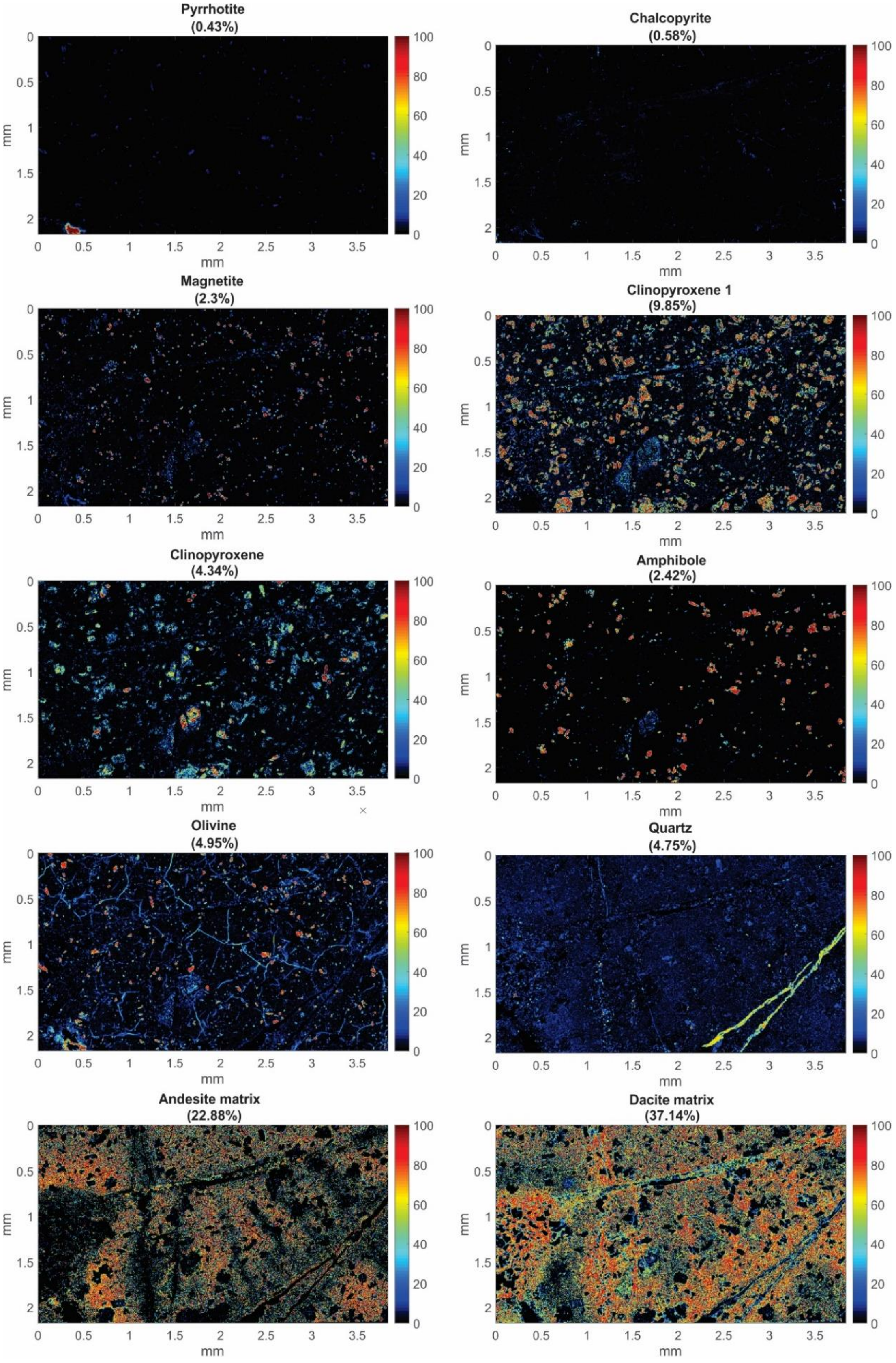

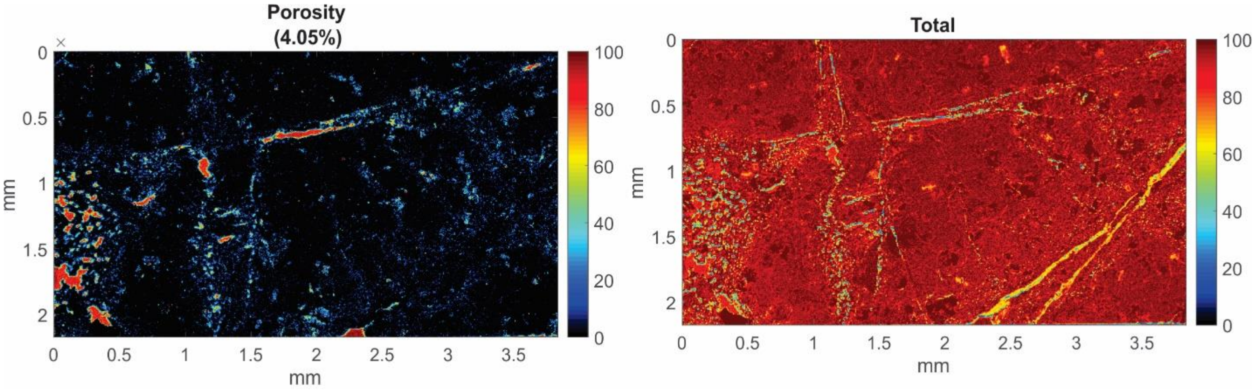

Supplementary figure 7. Mineral proportion maps calculated from elemental maps for sample TH-20-30-A. Ten phases are recognized in maps plus the porosity. The scale represents the percent of the specific phase at each spot analysis while the percent value below the phase name corresponds to the total phase fraction in the map.

Supplementary Figure 8

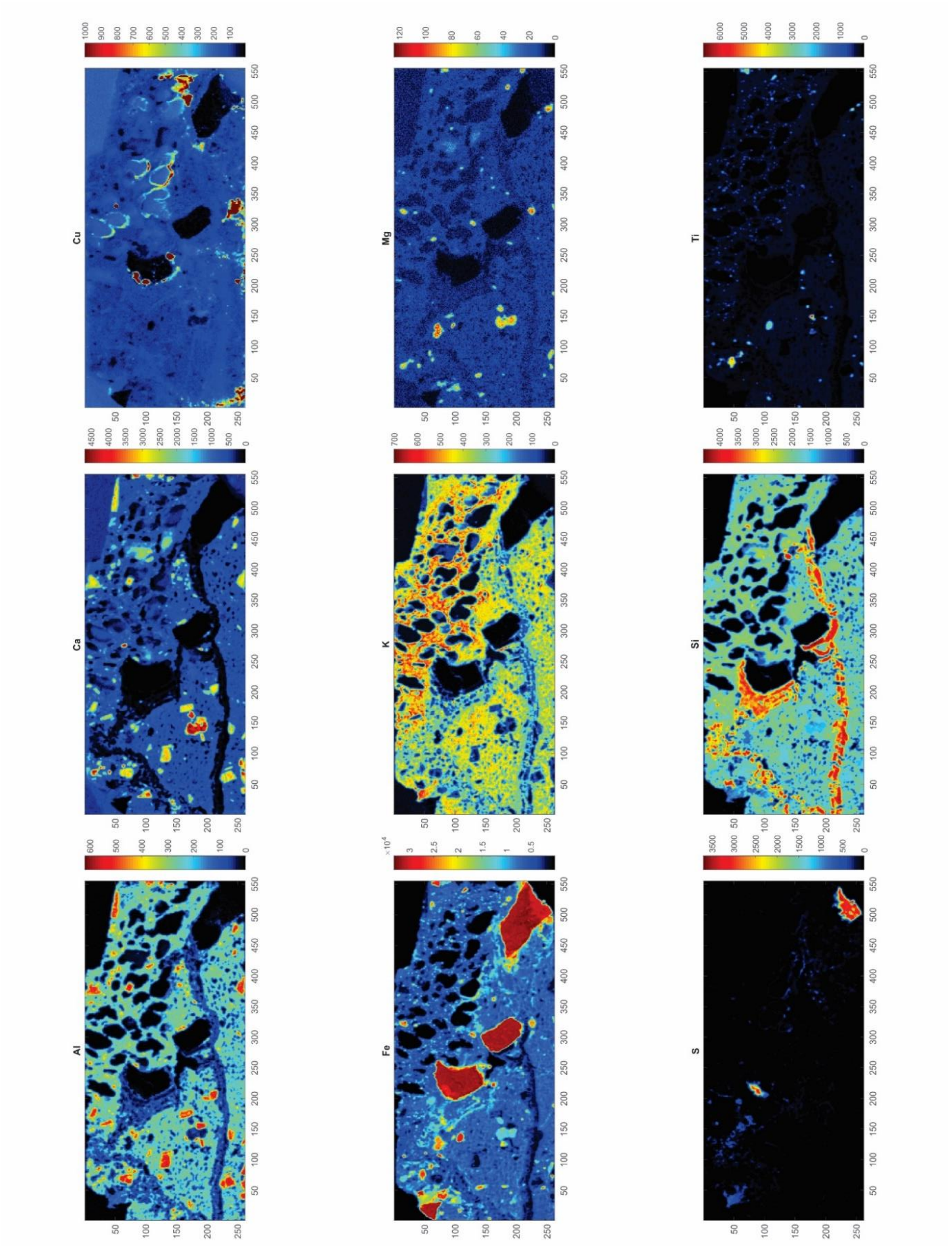

Supplementary figure 8. Chemical maps of Al, Ca, Cu, Fe, K, Mg, S, Si and Ti from sample TH-20-30-2 used for calculation of phase proportions by selecting regions of interests. The scales are absolute counts.

Supplementary Figure 9

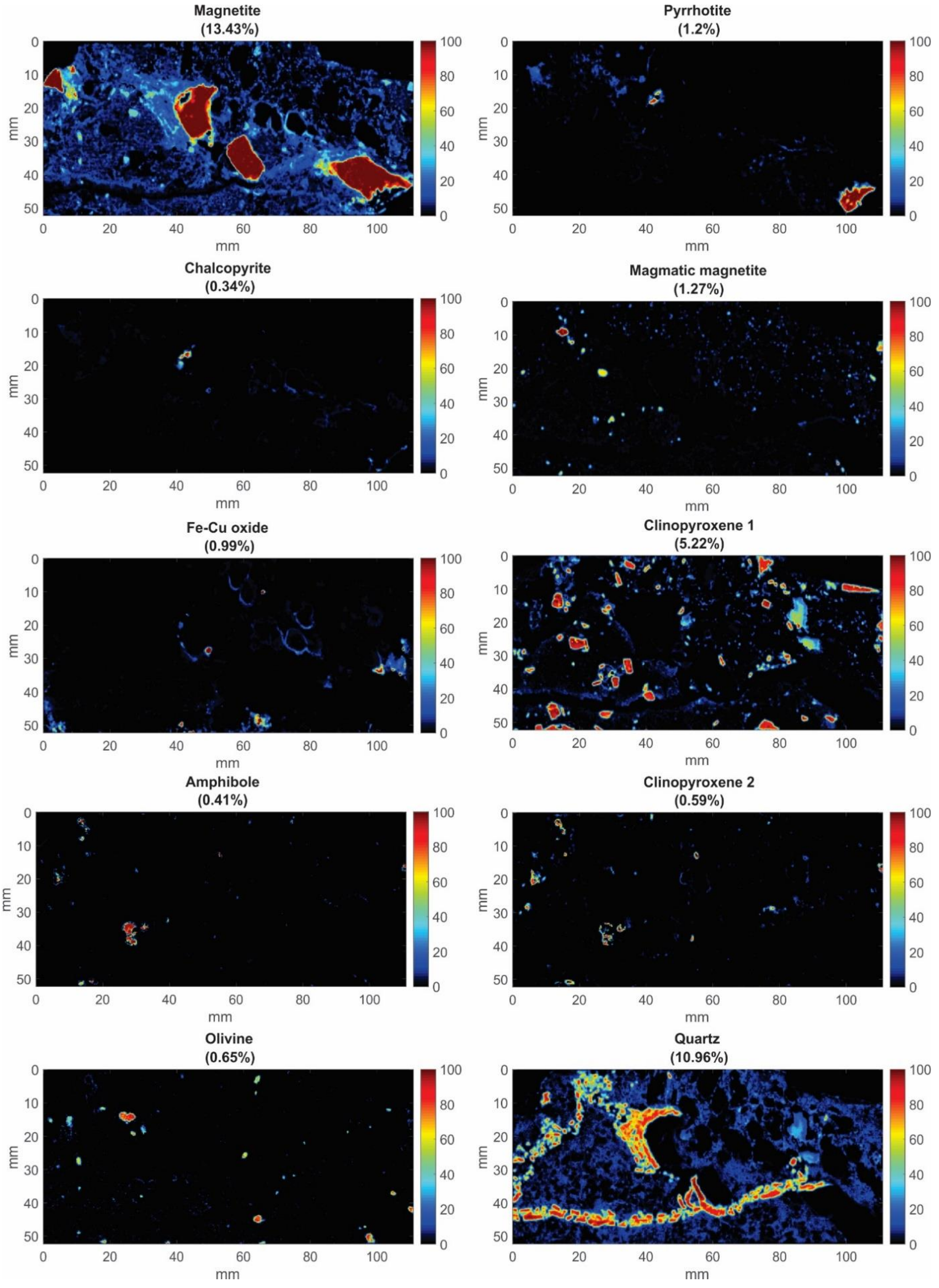

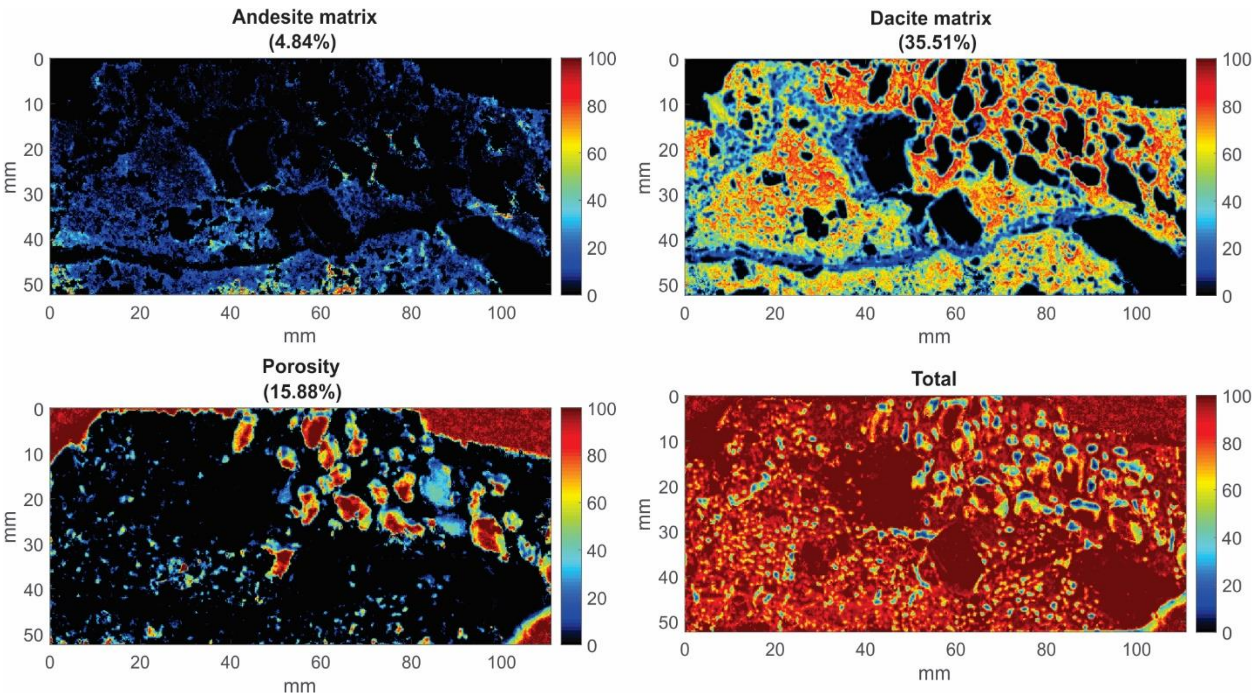

Supplementary figure 9. Mineral proportion maps calculated from elemental maps for sample TH-20-30-2. Thirteen phases are recognized in maps plus the porosity. The scales represent the percent of the specific phase at each spot analysis while the percent value below the phase corresponds to the total phase fraction in the map.

References

1. Peach, C. L., Mathez, E. A. & Keays, R. R. Sulfide melt-silicate melt distribution coefficients for noble metals and other chalcophile elements as deduced from MORB: Implications for partial melting. *Geochim. Cosmochim. Acta* **54**, 3379–3389 (1990).

2. Patten, C. G. C., Barnes, S.-J. & Mathez, E. A. Textural variations in morb sulfide droplets due to differences in crystallization history. *Can. Mineral.* **50**, 675–692 (2012).

3. Dare, S. A. S., Barnes, S. J. & Beaudoin, G. Variation in trace element content of magnetite crystallized from a fractionating sulfide liquid, Sudbury, Canada: Implications for provenance discrimination. *Geochim. Cosmochim. Acta* **88**, 27–50 (2012).

4. Czamanske, G. K. & Moore, J. G. Composition and phase chemistry of sulfide globules in basalt from the Mid-Atlantic Ridge rift valley near 37 N lat. *Geol. Soc. Am. Bull.* **88**, 587–599 (1977).

5. Edmonds, M., Mather, T. A. & Liu, E. J. A distinct metal fingerprint in arc volcanic emissions. *Nat. Geosci.* **11**, 790–794 (2018).

6. Lambert, G., Le Cloarec, M. F., Ardouin, B. & Le Roulley, J. C. Volcanic emission of radionuclides and magma dynamics. *Earth Planet. Sci. Lett.* **76**, 185–192 (1985).

7. Guo, H. & Audétat, A. Transfer of volatiles and metals from mafic to felsic magmas in composite magma chambers: an experimental study. *Geochim. Cosmochim. Acta* **198**, 360–378 (2017).

8. Dare, S. A. S. *et al.* Trace elements in magnetite as petrogenetic indicators. *Miner. Depos.* **49**, 785–796 (2014).
