# Supplementary material for: Transfer of sulfur and chalcophile metals via sulfide-volatile compound drops in the Christiana-Santorini-Kolumbo volcanic field
Source: Nat Commun. 2024 Jun 11;15:4968. doi: 10.1038/s41467-024-48656-9 (PMC11167051; doi:10.1038/s41467-024-48656-9)
Supplement: Supplementary file 3 — Description of Additional Supplementary Files [file 41467_2024_48656_MOESM3_ESM.pdf]

# <u>Description of Additional Supplementary Files</u>

**Supplementary Data 1:** Major element concentration of samples hosting sulfides and oxides related to compound drops.

<u>Description:</u> XRF analyses using a WDS Bruker AXS

**Supplementary Data 2:** Sulfide and oxide trace element concentrations.

<u>Description:</u> *Fe57 data from EMPA ** Fe internal standard for magmatic bleb is calculated from modal proportion of pyrrhotite, chalcopyrite and magnetite present in the magmatic sulfide blebs using equation (1) of the supplementary discussion and following Nadeau et al. (2010) approach. ***Partly oxidized chalcopyrite do not have stochiometric concentration and correspond locally to chalcopyrite-pyrite or covellite-pyrite mixture. Data below the limit of detection are not discarded but half of the detection limit value is used instead.

**Supplementary Data 3:** Magmatic volatile-sulfide emanation coefficients

<u>Description:</u> See Supplementary Discussion for emanation coefficient calculations

**Supplementary Data 4:** LA-ICP-MS reference material concentrations

<u>Description:</u> *No working values for Th in sulfide standards so the calibration is done using the glass standards BCR-2G, BHVO-2G and BIR-1G

**Supplementary Data 5:** Single spot LA-ICP-MS analyses

<u>Description:</u> Analyses below the limit of detection are reported in italic as half the value of the limit of detection. Fe data from EMPA are used as internal standard. n.d. not determined. LOD=limit of detection. Limit of detections calculated from Iolite using the method from Petkke et al. (2012). Pettke, T., Oberli, F., Audétat, A., Guillong, M., Simon, A. C., Hanley, J. J., & Klemm, L. M. (2012). Recent developments in element concentration and isotope ratio analysis of individual fluid inclusions by laser ablation single and multiple collector ICP-MS. Ore Geology Reviews, 44, 10-38.

**Supplementary Data 6:** ICP-MS analysis of mineralized samples from Kolumbo hydrothermal field
